# Correlation between salivary estrogen levels and oral epithelial cytokeratin 5 expression

**DOI:** 10.12688/f1000research.22536.2

**Published:** 2020-04-22

**Authors:** Juni Handajani, Nuraini Effendi, Wihaskoro Sosroseno

**Affiliations:** 1 Department of Oral Biology, Faculty of Dentistry, Universitas Gadjah Mada, Yogyakarta, 55281, Indonesia; 2 Faculty of Dentistry, Universitas Gadjah Mada, Yogyakarta, 55281, Indonesia; 3 Faculty of Dentistry, AIMST University, Bedong, Kedah, 08100, Malaysia

**Keywords:** Cytokeratin 5, Epithelial cells, Estrogen, Saliva

## Abstract

**Background:** Estrogen expression levels may be associated with age and may affect keratinization of the hard palate. Keratinized epithelium expresses cytokeratin 5 and 14 in the basal layer. The aim of this study was to determine the correlation between the levels of salivary estrogen and number of cytokeratin 5-positive oral epithelial cells.

**Methods:** A total of 30 female subjects were recruited and divided into children, adults and elderly (N=10 per group). Salivary estrogen levels and cytokeratin 5-expressing oral epithelial cells were assessed using ELISA and immunohistological methods, respectively. Data were analyzed using ANOVA with post hoc LSD test and Pearson’s correlation coefficient.

**Results:** The results showed that both the number of cytokeratin 5-positive cells and the level of salivary estrogen were significantly higher in adults but decreased in the elderly, as compared with those in children (p<0.05). Furthermore, the levels of salivary estrogen were significantly correlated with the number of cytokeratin 5-positive cells (r=0.815). The ANOVA result showed significance difference cytokeratin 5 expression and estrogen level (p<0.05). The post hoc LSD test revealed cytokeratin 5 expression and estrogen level to be significantly different in children, adults, and elderly participants (p<0.05).

**Conclusions:** These results suggest that the profile of salivary estrogen and oral epithelial cell-expressed cytokeratin 5 may be positively correlated with age and depend on age.

## Introduction

Estrogens are known to be involved in both the female and male reproductive systems, as well as both physiological and pathological regulation of cells and tissues ^1^. Estrogens consist of three major forms, estrone, estradiol and estriol, and function via two distinct nuclear receptors, estrogen receptor (ER)-α and ER-β ^1^. Estrogens are able to stimulate proliferation of basal epithelial cell and differentiation epithelium, leading to up-regulated epithelial keratinization ^2^. Since ER-β is expressed on human oral epithelium ^3^, estrogens clearly play a crucial role in both the physiology and pathology of oral epithelium. Measurement of salivary estrogen levels is, therefore, useful to detect the individual systemic or oral condition ^4^.

Cytokeratins, consisting of 19 keratins which are classified into type I (acidic) and type II (basic to neutral) keratin, are basic structural components of epithelial cells ^5^. Cytokeratin expression is abundant on oral epithelium and salivary glands during odontogenesis ^6, 7^. Along with the expression of cytokeratin 14, the palatal epithelium during palatogenesis expresses cytokeratin 5 ^8, 9^. Increased cytokeratin 5-positive cells in oral mucosa were also observed in traditional batik (dye) workers and might be due to continuous exposure to azo colour dyes ^10^. Since the levels of estrogen may be associated with oral epithelial keratinization ^2^, the aim of the present study was to assess whether the levels of salivary estrogen expression are statistically correlated with the number of cytokeratin 5-positive oral epithelial cells.

## Methods

This study was done on July to September 2016. Using Federer ^11^, the sample size formula was as follows:

(t-1) (n-1) > 15

Where t = number of groups and n = number of subjects per group. Number of groups (t) in this study was 3 so minimal number of subjects per group (n) were 8.5. In this study, the sample size was 30 participants, with the choice of 10 participants per group was a sample of convenience. We recruited subjects in person from Sleman District, Yogyakarta, Indonesia and mitigated bias by asking subjects to fill out a questionnaire. Subjects who met inclusion criteria were asked to sign informed consent.

### Participants

Subjects were 30 females divided into three groups, i.e., children (8–10 years old), adult (20–30 years old), and elder (>60 years old), with 10 subjects per group. The inclusion criteria were good oral hygiene status (assessed using the Oral Hygiene Index, developed by Greene and Vermillion) ^12^ and general health, not taking medications such as steroid, phenytoin, nifedipine, cyclosporine; not wear dentures or orthodontic appliance. Subjects approval was obtained by signing an informed consent (parents of children provided written informed consent). The protocol was approved by the Ethical Committee of the Faculty of Dentistry-Universitas Gadjah Mada (No. 00696/KKEP/FKG-UGM/EC/2016).

### Assessment of salivary estrogen levels

Saliva was collected from using non-stimulating method in the afternoon (16:00–18:00) for 1 minute. Saliva sample was stored in microtubes at -20°C prior to assay. Levels of salivary estrogen were measured using an ELISA kit (Catalog No. SLV-4188; DRG Salivary Estradiol, Germany) once for each participant.

### Staining of cytokeratin 5-positive cells

Epithelial cells of the hard palate mucosa were swabbed using a cytobrush, smeared on the glass slide, fixed in methanol-acetate solution (3:1) and then kept at 4°C before staining. Slides were stained using rapid, economical, acetic acid, papanicolaou (REAP) method to identify oral epithelial cells. Slide were put in 1% acetic for 10 seconds then in Harri’s hematoxylin at 60°C for 1 minute, in Orange G-6 and EA-50 for 1 minute respectively ^13^. Cytokeratin 5-positive cells were identified using an ABC staining kit (ImmunoCruz, Santa Cruz Biotechnology, USA). Following blocking with 1.5% blocking serum in PBS for 1 hour, the slides were then incubated overnight in a humidified chamber at 4°C with mouse monoclonal anti-cytokeratin 5 antibody (Thermo Fisher Scientific Cat# MA5-15347, RRID:AB_11009375, Thermo Fisher Scientific, USA) diluted in 1:500, immunoreacted with biotinylated secondary antibodies from a solution of 75 µl normal blocking serum stock, 5 ml PBS and 25 µl biotinylated secondary antibody stock (ImmunoCruz ^TM^, Mouse ABC Staining System Catalog No. sc-2017 Santa Cruz Biotechnology, RRID:SCR_008987, USA) at room temperature for 30 minutes, incubated with the avidin-biotin at room temperature for 30 minutes and visualized using an ABC staining system as described by the manufacturer (Catalog No. sc-2017, Santa Cruz Biotechnology, USA). The slides were then counterstained with methylene blue and viewed under a light microscope. The number of cytokeratin 5-positive cells was counted 100 epithelial cells in 10 fields per participant.

### Statistical analysis

Data obtained on salivary estrogen level and cytokeratin 5-positive cell expression were analyzed using ANOVA and Pearson’s correlation analysis. Following ANOVA, a post hoc least significant difference test was used to analyze differences in cytokeratin 5 expression and estrogen levels in children, adults and the elderly. Analysis was performed using IBM SPSS Statistics v22. P<0.05 was considered to indicate a statistically significant difference.

## Results

**Figure 1.**
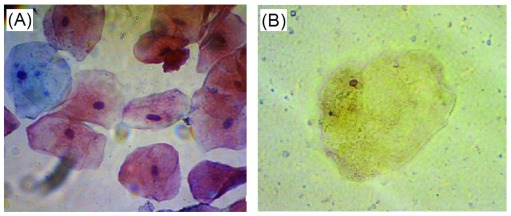
Palatal epithelial cells stained by Papanicolaou (x400) ( **A**) and immunohistologically stained for cytokeratin 5 ( **B**) (x400). Basal cells are stained as blue color. Intermediate and superficial are stained as pink and orange color, respectively. Cytokeratin 5 protein is stained as brown color in nucleus and cytoplasm.

**Figure 2.**
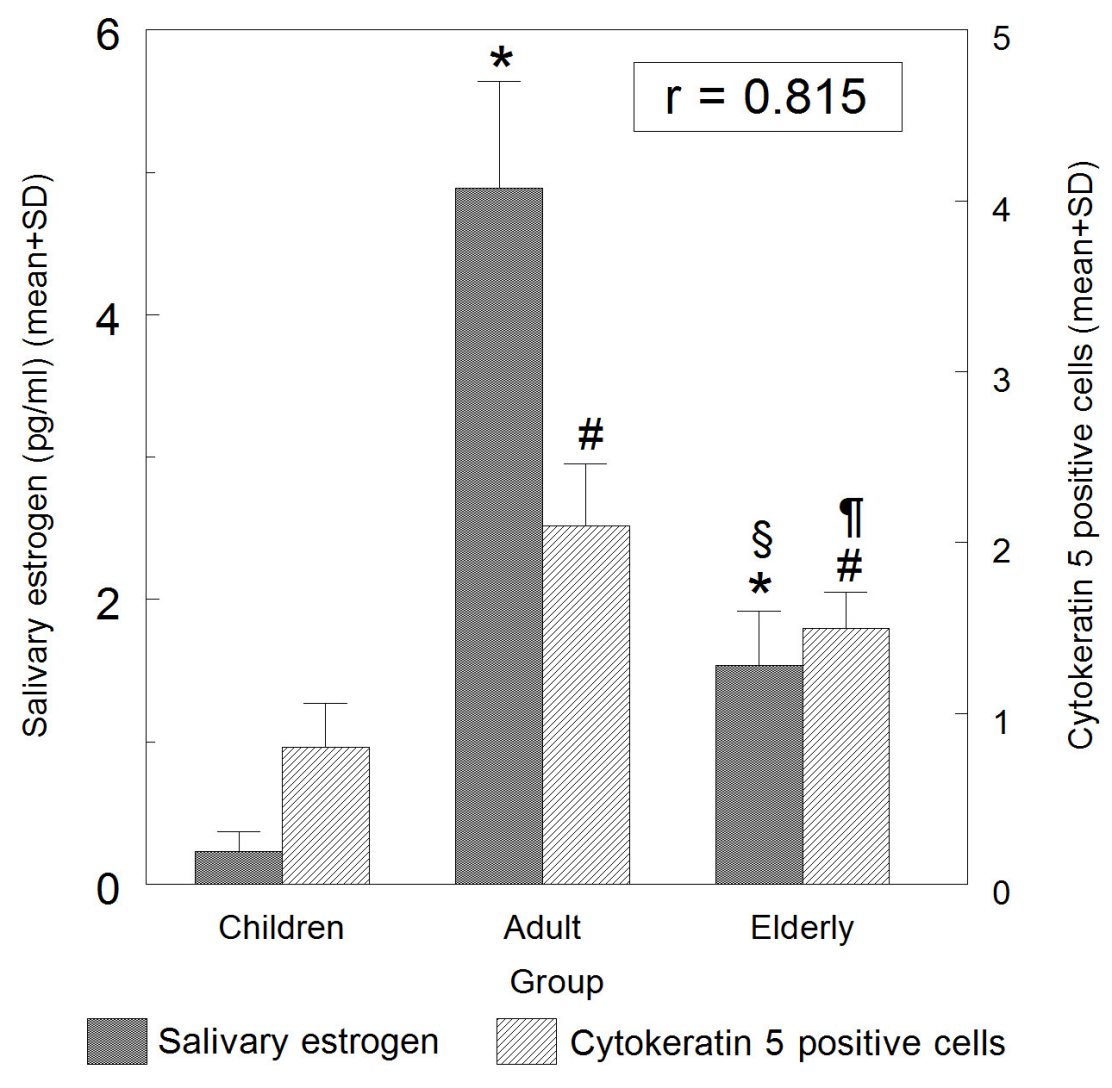
The correlation between the levels of salivary estrogen and cytokeratin 5-positive oral epithelial cells. *p<0.05 vs. the group of children at; § p<0.05 vs the group of adults at; #p<0.05 vs the group of children at; ¶p<0.05 vs the group of adults at.

The palatal epithelial cells stained by Papanicolaou and anti-cytokeratin 5 are depicted in Figure 1. The cytokeratin 5 positive staining could be observed in the cell nucleus and cytoplasm of basal, intermediate or superficial cell. As seen in Figure 2, salivary estrogen levels and cytokeratin 5-positive oral epithelial cell numbers were highest in adults, followed by the elderly, and then children (p<0.05). Based on Pearson’s correlation analysis, there was a positive correlation between salivary estrogen levels and the number of cytokeratin 5-positive cells (r = 0.815). Individual-level results are available as *Underlying data* ^14^.

Results of ANOVA are displayed in Table 1. Post hoc LSD test showed significance difference all comparison both cytokeratin 5 expression and estrogen levels ( Table 2)

**Table 1.** Results of ANOVA of cytokeratin 5 expression and estrogen level in children, adults, and the elderly.

Variable	n	F	P-value
Cytokeratin 5 expression	30	49.532	<0.001
Estrogen level (pg/ml)	30	236.409	<0.001

**Table 2.** Post hoc LSD result of cytokeratin 5 expression and estrogen level in children, adult, and elderly.

Cytokeratin 5 expression	Children	Adults	Elderly
Children	-	<0.001	<0.001
Adults	-	-	<0.001
Elderly	-	-	-
Estrogen level (pg/ml)	Children	Adults	Elderly
Children	-	<0.001	<0.001
Adults	-	-	<0.001
Elderly	-	-	-

## Discussion

The results in the present study showed that the highest salivary estrogen levels were found in adults, followed by the elderly and then children. These results correspond with a previous report that age changes affect hormone changes produced by the body ^15^. Almost no gonadotrophin hormone is secreted in children, hence the ovary remains inactive ^16^. Entering adult phase, increased estrogen levels are observed during the menstruation cycle at 24 hours before ovulation. However, estrogen levels are significantly decreased in aging ^16, 17^. The number of oral epithelial cells expressing cytokeratin 5 in adults was much higher than that in children and the elderly. The exact reason for these results remains to be studied. However, it is possible that increased cytokeratin 5-positive oral epithelial cells in adults may be due to the action of estrogens, which in turn may stimulate cell divisions on the basal layer and hence keratinization ^5^. Indeed, statistical analysis conducted in the present study indicated that increased number of cytokeratin 5-expressing oral epithelial cells is positively correlated with increased levels of salivary estrogen. That the main estrogen function is to increase cell proliferation and differentiation and tissue homeostasis ^1^ supports the results of present study.

The implication of the present study in the physiology and pathology of oral epithelial cells is yet to be determined. Alam and colleagues demonstrated that cytokeratin 5, along with cytokeratin 14, may function to maintain cell proliferation and differentiation in the basal layer of stratified epithelia ^18^, suggesting that increased levels of estrogen and number of cytokeratin 5-positive oral epithelial cells in adult females may be associated with promotion and maintenance of oral epithelial cell proliferation and differentiation.

In conclusion, the present study showed that the levels of salivary estrogen and the number of cytokeratin 5-positive oral epithelial cells in adult females are significantly higher than those in the children and elderly (p<0.05). The levels of salivary estrogen were strongly correlated with the number of cytokeratin 5-positive cells (r = 0.815). Therefore, the results of the present study suggest that the levels of salivary estrogen and the number of cytokeratin 5-positive oral epithelial cells may be an age-dependent phenomenon and are positively correlated.

## Data availability

### Underlying data

Figshare: Result estrogen-expresi cytokeratin 5-raw.xlsx. https://doi.org/10.6084/m9.figshare.11888727.v4 ^14^.

This project contains the following underlying data:

- Result estrogen-expresi cytokeratin 5-raw (XLSX). Salivary estrogen levels and number of cytokeratin-5-positive cells for each participant.
- Raw image files used for each figure (JPG).

Data are available under the terms of the Creative Commons Attribution 4.0 International license (CC-BY 4.0).
