# Supplementary material for: Correlation between salivary estrogen levels and oral epithelial cytokeratin 5 expression
Source: F1000Res. 2020 Apr 22;9:186. Originally published 2020 Mar 12. [Version 2] doi: 10.12688/f1000research.22536.2 (PMC7194496; doi:10.12688/f1000research.22536.2)
Supplement: Peer review history

# Peer review: Reviewer response for version 2

**DOI:** 10.5256/f1000research.25912.r62622

**Published:** 2020-04-23

**Authors:** Hirohiko Okamura

**Affiliations:** 1 Department of Oral Morphology, Graduate School of Medicine, Dentistry and Pharmaceutical Sciences, Okayama University, Okayama, Japan

I have no further comments.

Is the work clearly and accurately presented and does it cite the current literature?

Yes

If applicable, is the statistical analysis and its interpretation appropriate?

I cannot comment. A qualified statistician is required.

Are all the source data underlying the results available to ensure full reproducibility?

Yes

Is the study design appropriate and is the work technically sound?

Yes

Are the conclusions drawn adequately supported by the results?

Yes

Are sufficient details of methods and analysis provided to allow replication by others?

Yes

Reviewer Expertise:

bone metabolism; transcriptional regulation; extracellular vesicles

I confirm that I have read this submission and believe that I have an appropriate level of expertise to confirm that it is of an acceptable scientific standard.

# Peer review: Reviewer response for version 1

**DOI:** 10.5256/f1000research.24873.r61608

**Published:** 2020-04-07

**Authors:** Euis Reni Yuslianti

**Affiliations:** 1 Department of Biochemistry and Molecular Biology, Faculty of Medicine, Jenderal Achmad Yani University, Cimahi, Indonesia

Dr Handajani and collagues have worked clearly and accurately presented. The study design,  statistical analysis, and its interpretation is appropriate with the methods to allow replication by other researchers. This article have good implication to explain the correlation between turn over cell in females oral mucosa with salivary estrogen levels. The discussion would be better and more deep if researchers explained about the correlation between hormonal mechanism, especially estrogen, with oral mucosa cell cycles and aging in human being.

Is the work clearly and accurately presented and does it cite the current literature?

Yes

If applicable, is the statistical analysis and its interpretation appropriate?

Yes

Are all the source data underlying the results available to ensure full reproducibility?

Yes

Is the study design appropriate and is the work technically sound?

Yes

Are the conclusions drawn adequately supported by the results?

Yes

Are sufficient details of methods and analysis provided to allow replication by others?

Yes

Reviewer Expertise:

oral biology, biochemistry, aging, free radical and antioxidant

I confirm that I have read this submission and believe that I have an appropriate level of expertise to confirm that it is of an acceptable scientific standard.

# Peer review: Reviewer response for version 1

**DOI:** 10.5256/f1000research.24873.r61298

**Published:** 2020-03-23

**Authors:** Hirohiko Okamura

**Affiliations:** 1 Department of Oral Morphology, Graduate School of Medicine, Dentistry and Pharmaceutical Sciences, Okayama University, Okayama, Japan

In this paper entitled “Correlation between salivary estrogen levels and oral epithelial cytokeratin 5 expression”, Handajani *et al.*, mainly examined the relationship salivary estrogen levels and cytokeratin 5 expression in oral epithelial cells. Salivary estrogen levels in adult group was higher than that of children group, and decreased in aging. In parallel, the number of cytokeratin 5-positive oral epithelial cells was higher in adult group compared with other groups. By using statistical analysis, the authors concluded that there is a positive correlation between the levels of salivary estrogen and the number of cytokeratin 5-positive oral epithelial cells, which may be age-dependent phenomenon. The following points should be addressed for considering indexing in the journal “ *F1000Research".*

 Major comments;

1. In fig. 1, they showed two pictures (A) and (B) with same magnification (x400). However, there are about 10 cells can be observed in (A), whereas only single cell in (B). Why? To prove the reliability of experiment (Cytokeratin 5 staining), please show the picture, in which at least 2-3 cells can be observed.
2. Are there any differences in cytokeratin 5 expression between basal cells and intermediate and superficial cells?
3. In this study, the authors examined salivary estrogen levels and cytokeratin 5 expression in only female group. Why did the authors exclude male participants? Are there any reasons?
4. Even in the highest group (adult) of cytokeratin 5, about 2 cells were positive. Other cells are all negative? How about the expressions of other types of cytokeratin?

Is the work clearly and accurately presented and does it cite the current literature?

Yes

If applicable, is the statistical analysis and its interpretation appropriate?

I cannot comment. A qualified statistician is required.

Are all the source data underlying the results available to ensure full reproducibility?

Yes

Is the study design appropriate and is the work technically sound?

Yes

Are the conclusions drawn adequately supported by the results?

Yes

Are sufficient details of methods and analysis provided to allow replication by others?

Yes

Reviewer Expertise:

bone metabolism; transcriptional regulation; extracellular vesicles

I confirm that I have read this submission and believe that I have an appropriate level of expertise to confirm that it is of an acceptable scientific standard, however I have significant reservations, as outlined above.

# Response

**Published:** 2020-04-08

**Authors:** Juni Handajani

**Affiliations:** Universitas Gadjah Mada, Indonesia

1. In fig. 1, they showed two pictures (A) and (B) with same magnification (x400). However, there are about 10 cells can be observed in (A), whereas only single cell in (B). Why? To prove the reliability of experiment (Cytokeratin 5 staining), please show the picture, in which at least 2-3 cells can be observed.

 Answer :

 The raw data was showed in Result estrogen-ekspresi cytokeratin 5-raw.xlsx. https://doi.org/10.6084/m9.figshare.11888727.v118. One figure showed 2-3 positive cells of cytokeratin 5.

 2. Are there any differences in cytokeratin 5 expression between basal cells and intermediate and superficial cells?

 Answer :

 Cytokeratin 5 positive cell was expressed brown color, we couldn't identify cytokeratin 5 positive cell expression from basal or intermediate or superficial cell 

 3. In this study, the authors examined salivary estrogen levels and cytokeratin 5 expression in only female group. Why did the authors exclude male participants? Are there any reasons?

 Answer :

 Estrogen may have role in differentiation and keratinization in oral epithelial cell. Some study showed that estrogen hormone in female higher than male.

 4. Even in the highest group (adult) of cytokeratin 5, about 2 cells were positive. Other cells are all negative? How about the expressions of other types of cytokeratin?

 Answer :

 We counted the number of cytokeratin 5-positive cells per 100 epithelial cells in 10 fields per participant, the other cells are negative expression. We didn't analyze other type of cytokeratin yet.
